# Insulin Directs Dichotomous Translational Regulation to Control Human Pluripotent Stem Cell Survival, Proliferation and Pluripotency

**DOI:** 10.7150/ijbs.71199

**Published:** 2022-05-16

**Authors:** Xiaoxiao Zhou, Zhili Ren, Jiaqi Xu, Chunhao Deng, Zhaoying Zhang, Carlos Godoy-Parejo, Faxiang Xu, Esther Chi Cheng Huang, Jiajia Wang, Zheyu Cai, Weiwei Liu, Guang Hu, Guokai Chen

**Affiliations:** 1 Centre of Reproduction, Development and Aging, Faculty of Health Sciences, University of Macau, Macau SAR, China.; 2 Epigenetics and Stem Cell Biology Laboratory, National Institute of Environmental Health Sciences, RTP, NC 27709, USA.; 3 Animal Research Core Facility, Faculty of Health Sciences, University of Macau, Macau SAR, China.; 4 Institute of Translational Medicine, Faculty of Health Sciences, University of Macau, Macau SAR, China.; 5 Bioimaging and Stem Cell Core Facility, Faculty of Health Sciences, University of Macau, Macau SAR, China.; 6 MoE Frontiers Science Center for Precision Oncology, University of Macau, Macau SAR, China.

**Keywords:** Cell Survival, Insulin, AKT, NOXA/PMAIP1, mTOR, eIF4E, Pluripotency, Proliferation, Translation, Human pluripotent stem cell (hPSC)

## Abstract

Insulin is essential for diverse biological processes in human pluripotent stem cells (hPSCs). However, the underlying mechanism of insulin's multitasking ability remains largely unknown. Here, we show that insulin controls hPSC survival and proliferation by modulating RNA translation via distinct pathways. It activates AKT signaling to inhibit RNA translation of pro-apoptotic proteins such as NOXA/PMAIP1, thereby promoting hPSC survival. At the same time, insulin acts via the mTOR pathway to enhance another set of RNA translation for cell proliferation. Consistently, mTOR inhibition by rapamycin results in eIF4E phosphorylation and translational repression. It leads to a dormant state with sustained pluripotency but reduced cell growth. Together, our study uncovered multifaceted regulation by insulin in hPSC survival and proliferation, and highlighted RNA translation as a key step to mediate mitogenic regulation in hPSCs.

## Introduction

Human pluripotent stem cells (hPSCs) have unlimited self-renewal ability and can generate all cell types in our body 1. Continuous insulin stimulation is required to maintain fundamental stem cell activities such as cell survival, proliferation and pluripotency 2-5. It is intriguing how a single mitogen could coordinate diverse functional components to carry out all these concurrent activities. In the flow of genetic information from DNA to protein, translational regulation alone contributes more to the regulatory amplitude than the sum of other regulations including epigenetic modification, transcription, RNA degradation and protein degradation 6-8. Translation is theoretically an ideal target for multilaterial regulation by insulin in hPSCs, but little evidence is available.

Nearly every cellular process involves proteins, whose production is influenced by various mitogens. Upon mitogenic stimulation, translation regulation takes immediate effect to influence protein synthesis from existing mRNAs. The formation of eukaryotic initiation factor 4F complex (eIF4F) is the first stage of the cap-dependent translation, and it is also the chief target of translation regulation. eIF4F components are mainly regulated by the mammalian target of rapamycin (mTOR) and the mitogen-activated protein kinase (MAPK) pathway 9. mTOR integrates mitogenic and nutritional signals to elevate global translation via ribosomal protein S6 kinase (S6K) and eukaryotic translation initiation factor 4E (eIF4E) 10. The PI3K/AKT pathway is the best-known mTOR activator under mitogen stimulation 11. At the same time, MAPK pathway regulates eIF4E phosphorylation through MAPK interacting protein kinases (MNKs) 12. eIF4E phosphorylation is reported to either to promote or inhibit translation in a cell type specific manner 13, 14.

Stem cells have distinctive translation regulation from somatic cells 15. Both pluripotent and multipotent stem cells display lower translation rate than somatic cells. It is suggested that lower translation activities are necessary to maintain an undifferentiated state 16. When pluripotent stem cells differentiate, global translation increases, accompanied by elevated mTOR activity 16, 17. When mouse blastocytes are treated with mTOR inhibitor rapamycin, a paused pluripotent stage is induced that allows prolonged *ex vivo* embryo culture. Similar phenomenon is observed in naïve mouse pluripotent stem cells. 18. These observations imply that translation inhibition through mTOR pathway does not cause the loss of naïve pluripotency in mouse. In contrast, differentiation is induced in hPSCs by rapamycin under maintenance condition containing Knock-Out Serum Replacement (KOSR) 19, 20. Considering that hPSCs are at primed pluripotency state, it is still unclear whether the distinctive responses to mTOR inhibition are caused by the difference in either species or pluripotency state. Although transcriptome regulation is well reported, much less is known about translatome regulation on key functions in pluripotent stem cells.

During self-renewal, hPSCs have to survive and proliferate while maintaining pluripotency. In order to carry out these concurrent functions, both protein synthesis and translation inhibition are essential. For example, cell proliferation relies on continuous translation to produce proteins for metabolism and cell growth; in parallel, cell survival may demand translation inhibition of pro-apoptotic proteins. Insulin is not only the most important mitogen for cell survival but also necessary for proliferation 2, 3, so we hypothesize that insulin may orchestrate multiplex translation regulation to promote self-renewal. In order to examine the immediate translation regulation by signaling pathways, ribosome-associated mRNAs are analyzed to understand the global translation responses. We reveal that insulin triggers concurrent activation and inhibition of mRNA translation essential for cell survival and proliferation. Insulin activates AKT to suppress the synthesis of pro-apoptotic proteins such as NOXA/PMAIP1. Meanwhile, insulin promotes cell proliferation through mTOR-dependent translation. Furthermore, we demonstrate that hPSC pluripotency is decoupled from mTOR-related translation regulation. Although global translation is suppressed by mTOR inhibitor rapamycin, a dormant pluripotency state is established that is sensitive to differentiation induction. This study reveals that insulin simultaneously modulate fundamental stem cell activities through multiplex translation regulation, and highlights that translational inhibition is the key mechanism to establish insulin as one of the essential hPSC mitogens.

## Materials and Methods

### hPSC

Human embryonic stem cell (hESC) lines (H1, H9) and human induced pluripotent stem cell (iPSC) line NL4 from NIH were used in this project. Cells were cultured in E8 medium (DMEM/F12, L-ascorbic acid, selenium, transferrin, insulin, FGF2 and TGFβ) on matrigel coated surface for maintenance^2^ and passaged with EDTA/DPBS every 3-4 days following standard procedure described previously^21^. ROCK inhibitor Y-27632 (5 µM) was added on the day of cell passaging. For insulin removal, medium contains DMEM/F12, L-ascorbic acid, selenium, transferrin, FGF2 and TGFβ.

### Nude mice

Nude Mice used in this study were obtained from the Animal Facility in the Faculty of Health Sciences at the University of Macau. All procedures were performed in compliance with the Animal Care and Use Committee of the University of Macau.

### Cell survival and proliferation assay

The assay was performed as previously described with some modifications 22. Briefly, hESCs or iPSCs were dissociated with EDTA/DPBS and seeded into 24-well plates. Treatments were added on the next day. 48 hours later, cells were dissociated with TrypLE, neutralized with 0.5% BSA in DMEM/F12, and collected for counting. Cell counts were determined by a flow cytometer.

### Polysome profiling

Polysome profiling was performed as previously described with some modifications 23. Briefly, H1 cells at 50% confluency were subjected to insulin removal or other treatments as specified. Prior to harvesting, 100 μg/ml cycloheximide (Sigma-Aldrich, St. Louis, MO, USA) were added to cells and incubated for 10 min. Wash cells with ice-cold 1 × PBS + 100 μg/ml cycloheximide, scrap off, and centrifuge to get cell pellet. Each sample was lysed on ice for 30 min in 500 μl polysome lysis buffer containing 12.5 mM Tris pH 7, 7.5 mM Tris pH 8, 15 mM MgCl_2_, 200 mM NaCl, 1 mM DTT, 100 μg/ml cycloheximide, 1% TritonX-100, and 20 U/ml Superase Inhibitor (Ambion, #AM2696). Cell lysates were centrifuged at 13,000 rpm, 4ºC for 10 min. 10 to 60% RNase-free sucrose gradients (15 mM Tris pH 8, 100 mM KCl, 3 mM MgCl_2_, 1 mM DTT, 100 μg/ml cycloheximide and 20 U/ml Superase Inhibitor (Ambion, #AM2696)) were prepared by a BioComp Gradient maker (BioComp Instruments, Fredericton, NB, Canada) according to the user guide. 500 µl cell lysate was put on top of the sucrose gradient and centrifuged at 41,000 rpm, 4°C for 4 h. A BioComp Gradient Station with fractionator and optical monitor at a 260 nm wavelength was used to get the polysome profiles.

### Western blotting

Cells were lysed with Laemmli buffer, and the total protein concentrations were determined using BCA kit (Thermo Scientific, #23228). 20 µg total protein was loaded into each well for electrophoresis and transferred to PVDF membrane. Membrane was blocked in 5% non-fat milk in TBST, incubated with primary antibodies overnight at 4℃, washed, and incubated with HRP-conjugated secondary antibodies at room temperature for 2 h. SuperSignal West Pico Chemiluminescent Substrate (Thermo Fisher) was used to develop chemiluminescence and the signal was detected using ChemiDoc Imaging System (Bio-Rad).

### Ribosome-nascent chain complex (RNC) extraction

Total RNA and RNC-RNA extraction and sequencing were performed as previously described 24, 25. Total RNA was extracted from cultured cells using RNAiso Plus reagent (Takara, 9109) following manufacturer's instructions. For RNC-RNA extraction, cells were incubated with 100 μg/ml cycloheximide for 10 min, washed with ice-cold 1× PBS + 100 μg/ml cycloheximide, and then scraped off in 1× PBS +100 μg/ml cycloheximide and collected by centrifugation. Cell pellets were lysed on ice for 30 min in lysis buffer (1% Triton X-100 in ribosome buffer). Ribosome buffer contains 20 mM HEPES-KOH (pH 7.4), 15 mM MgCl_2_, 200 mM KCl, 100 μg/ml cycloheximide, and 2 mM dithiothreitol. The cell lysate was layered onto the surface of sucrose buffer (30% sucrose in ribosome buffer) followed by ultra-centrifugation at 185,000 x *g*, 4 °C for 5 h. RNCs were extracted from the pellet and RNC-RNA was purified with RNAiso Plus reagent.

### Strand-specific RNA-seq library construction and sequencing

The mRNA was purified from total RNA or RNC-RNA samples using magnetic beads with poly-T oligos attached. Libraries were generated using NEBNext^®^ UltraTM RNA Library Prep Kit for Illumina^®^ (NEB E7530) following manufacturer's protocol, and sequencing was performed using an Illumina Hiseq 4000 system, generating 150 bp paired-end reads. All sequencing datasets in this study are available at Sequence Read Archive (SRA) (accession number SUB8322997).

### Construction of knockdown cell lines

shRNA constructs shNOXA were obtained from Fulengen, Inc. pLKO.1-TRC control plasmid (Addgene) was used as negative control. Lentivirus packaging using psPAX2 and pMD2.G plasmids was performed in 293FT cells according to the Addgene protocol (https://www.addgene.org/protocols/plko/). hESCs transduced with the indicated viruses were selected using puromycin (20 μg/ml).

### Real time PCR

Total RNA was extracted from cells using RNAiso Plus reagent. cDNA was generated from 500 ng total RNA by reverse transcription using high-capacity cDNA reverse transcription kit (Thermo Fisher). Real time PCR was performed using SYBR^®^ Premix Ex Taq™ kit (Takara) on a QuantStudio™ 7 Flex Real-Time PCR System. Gene expression levels were normalized to GAPDH. See Table 1 for primer sequences.

### Immunocytochemistry

Cells were washed with PBS twice, fixed with 4% paraformaldehyde in PBS for 20 min, washed again and then permeabilized with 0.5% Triton-X 100 in PBS for 20 min. After washing with PBS, cells were blocked with 1% BSA in 0.1% Triton-X100 / PBS, incubated with the primary antibodies overnight at 4℃, washed again, and finally incubated with secondary antibodies for one hour at room temperature. For actin staining, diluted phalloidin solution (Thermo Fisher, 1:100) was added to the cells and incubated for 30 min at room temperature. Nuclei were stained with hoechst (1:10,000). Samples were mounted with Vectashield mounting medium (Vector Laboratories) and imaged with Carl Zeiss LSM710 confocal microscope.

### Teratoma formation

hESCs were cultured in the presence of rapamycin for 3 passages and harvested using the EDTA method at around 80% confluency. Cells were collected in E8 medium with 5 µM rock inhibitor Y27632, pelleted by centrifugation, and then resuspended in a 1:1 mixture of E8/Y27632 medium and Matrigel (BD Biosciences, 356234) to a total volume of 300 μl. Approximately 2 × 10^6^ cells were injected in the dorsolateral area of nude mice into the subcutaneous space. Teratomas were harvested after 6-8 weeks for histological analysis.

### Flowcytometric analysis

Cells were dissociated with TrypLE and neutralized with 0.5% BSA in DMEM/F12. After washed with PBS, cells were fixed with 4% paraformaldehyde in PBS for 20 min at room temperature and permeabilized with 0.5% Triton X-100 in PBS for 20 min at room temperature. Cells were then incubated with Alexa Fluor^®^ 488 Conjugated mouse anti-human OCT4 antibodies (MERCK, FCMAB124A4) at a 1:100 dilution in 1% BSA in PBS for 1 h at room temperature. After washing, cells were resuspended in PBS for flow cytometry analysis using BD Biosciences Accuri C6 flow cytometer.

### Lineage-specific differentiation

For differentiation towards mesoendoderm lineages, H1 cells were cultured in E8 medium without TGFβ, in the presence of BMP4 (20 ng/µl). RNA was harvested on day 3 for qPCR analysis of *TBXT* and *SOX17* expression. For differentiation towards ectoderm, H1 cells were cultured in E6 medium (E8 medium without FGF2 or TGFβ) in the presence of SB431542 (10 µM). RNA was harvested on day 4 for qPCR analysis of *PAX6* expression.

### Statistical analysis

Data are presented as mean ± SD of three independent experiments unless otherwise specified. Statistical significance was determined by unpaired, two-tailed Student's t-test using Microsoft Excel, and p < 0.05 was considered as statistically significant (*).

## Results

### Insulin removal causes both mTOR-dependent and independent changes in RNA translation

**Figure 1:**
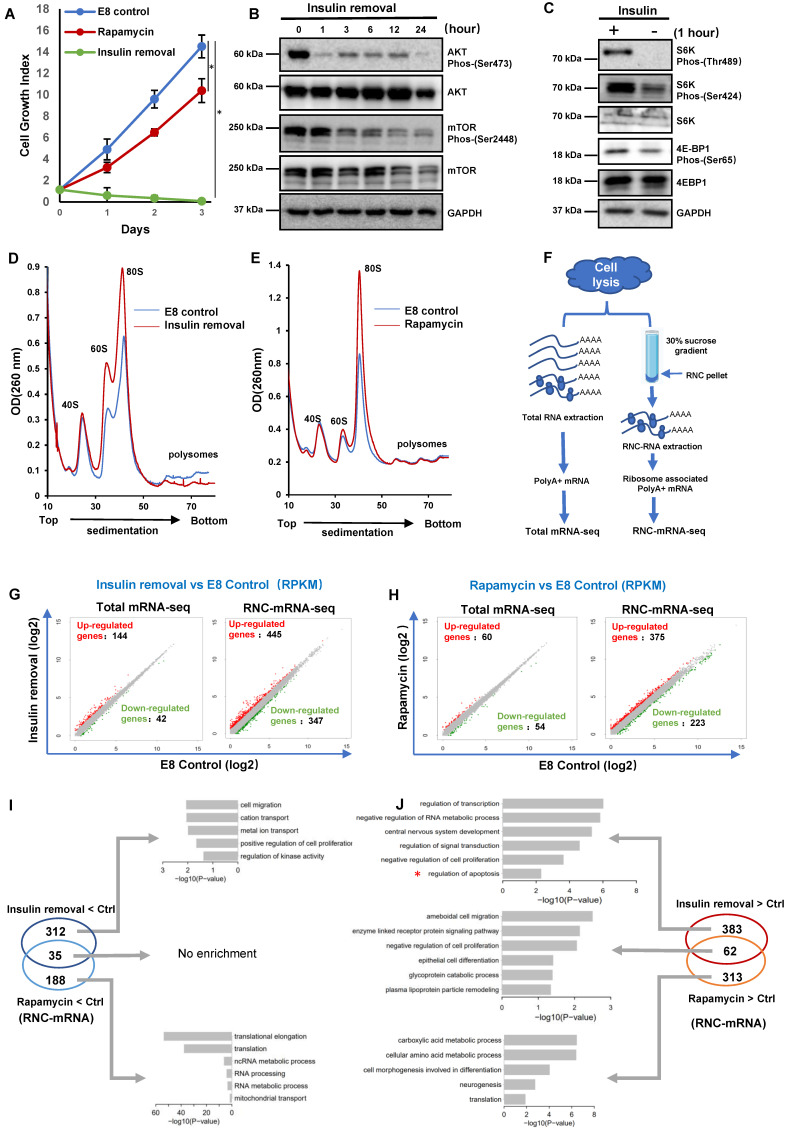
** Divergent translational regulation by insulin through AKT and mTOR. (A)** Growth numbers of H1 cells cultured in E8 control, E8 without insulin or E8 with rapamycin (100 nM). Cells were counted every day. Cell counts were normalized to day 0 numbers (n = 3 biological replicates, ∗, p < 0.05). **(B)** Western blot analysis of AKT and mTOR phosphorylation in H1 cells following insulin removal. Cells were cultured in E8 medium without insulin for 24 h. **(C)** Western blot analysis of S6K and 4E-BP1 phosphorylation in H1 cells one hour after insulin removal. Cells were cultured in E8 medium with or without insulin. GAPDH was used as loading control. **(D-E)** Polysome profiles of H1 cells cultured in E8 medium with or without insulin for 6 hours (D) or in E8 medium with or without rapamycin (100 nM) for 2 h (E). (Data are representative of two independent experiments). **(F)** Illustration of the full-length translating mRNA sequencing (RNC-seq) protocol. H1 cells were subjected to insulin removal or rapamycin treatment prior to cell lysis and total mRNA or RNC mRNA extraction. **(G-H)** Scatter plot of differentially expressed total mRNAs and RNC-mRNAs in H1 cells treated with insulin removal (G) or rapamycin (H) for 2h. x- and y-axes represent log2 transformed RNA expression value (RPKM). **(I-J)** Venn diagram illustrating the distribution of RNC-mRNAs regulated by 2-hour treatment of insulin removal or rapamycin. Gene Ontology (GO) term analysis of RNC-mRNAs upregulated both by insulin removal and by rapamycin treatment.

Insulin is essential for both hPSC survival and proliferation in chemically defined E8 medium, and it takes effect through PI3K/AKT pathway that is upstream of translation regulator mTOR 2, 3. In order to understand insulin's role in translation regulation, we used H1 hESCs to study the impact of insulin removal and rapamycin on hPSCs. In E8 medium, cells continuously proliferated in three-day time course. When insulin was removed from E8 (Insulin removal), cell growth was inhibited, and most cells died after 3 days. At the same time, rapamycin led to suppressed cell growth but without significant cell death (Figure 1A). We then showed that the cleavage of caspase-3 and caspase-7 was triggered by insulin removal but not by rapamycin (Figures S1A). These data suggested that insulin regulates both cell survival and proliferation beyond mTOR-associated translation.

To evaluate the connection between insulin and translation machinery, we examined key molecules downstream of insulin signaling. We showed that AKT phosphorylation decreased one hour after insulin removal, while mTOR phosphorylation also decreased gradually (Figure 1B). The phosphorylation of mTOR substrates, S6K and 4E-BP1, was suppressed significantly one hour after insulin removal (Figure 1C). These data demonstrated that insulin exerted immediate influence on translation regulation, at least partially through mTOR regulation.

To assess the translational responses caused by insulin removal and mTOR inhibition, we compared the polysome profiles of cells under different treatments. Six hours after insulin removal, the abundance of the 60S ribosomal subunits as well as the 80S monosomes was increased a lot, but the abundance of the polysomes was decreased (Figure 1D). In contrast, rapamycin treatment only caused an increase in the abundance of the 60S and 80S monosomes, but had no impact on the polysomes (Figure 1E). These results indicated that both insulin removal and rapamycin suppress mRNA translation, they had different effects on hPSCs, consistent with our observations in proliferation and survival (Figure 1A-C).

To further investigate the consequence of insulin removal and mTOR inhibition on translation, we performed regular RNA sequencing (Total mRNA-seq) and ribosome nascent-chain complex-bound RNA sequencing (RNC-mRNA-seq) (Figure 1F). Upon insulin removal, 144 transcripts were up-regulated and 42 were down-regulated at the RNA level based on total mRNA-seq. However, in RNC-mRNA-seq 347 transcripts showed increased association with the ribosomes and 445 showed decreased association (Figure 1G), suggesting that insulin removal induced profound changes in mRNA translation. Similar to insulin removal, rapamycin also had limited impact at the total RNA level, but more profound changes in ribosome-associated RNAs (Figure 1H). These data suggested that hESCs undergo greater changes in mRNA translation than transcription in their initial responses to insulin removal and rapamycin.

We then examined ribosome-associated mRNAs that were specifically affected by different treatments. The 347 ribosome-associated mRNAs which were decreased after insulin removal were enriched for genes involved in cytokine receptor interaction and purine metabolism (Figure S1B). The 445 ribosome-associated mRNAs that were increased after insulin removal were enriched for genes involved in signal transduction, including WNT, TGFβ and P53 pathways (Figure S1C). Meanwhile, the 223 ribosome-associated mRNAs downregulated after rapamycin treatment were involved in ribosome activity (Figure S1D), and those upregulated 375 mRNAs were involved in cell adhesion, ECM receptors interaction and amino acid metabolism (Figure S1E).

Furthermore, we showed that 35 ribosome-associated mRNAs were decreased by both insulin removal and rapamycin, and there was no obvious enrichment in specific processes (Figure 1I). 312 ribosome-associated mRNAs were only decreased by insulin removal, and they were enriched in positive regulation in cell proliferation, kinase activity, ion transportation. Rapamycin exclusively downregulated 188 ribosome-associated mRNAs that were related to translation, RNA processing and mitochondrial activity (Figure 1I). Surprisingly, the decreased ribosome-associated mRNAs did not reflect cell death phenotype after insulin removal and growth arrest by rapamycin, so we inspected ribosome-associated mRNAs that were increased by the treatments (Figure 1J). 62 ribosome-associated mRNAs were increased by both insulin removal and rapamycin, and they were enriched in negative regulation of cell proliferation, enzymes and glycogen metabolism. 383 ribosome-associated mRNAs were only increased by insulin removal, and they were enriched in negative regulation in cell proliferation, apoptosis, signal transduction and RNA processes. Rapamycin exclusively upregulated 313 ribosome-associated mRNAs that were related to translation, metabolism and morphogenesis. These data implied that insulin directed concurrent hESC translation regulation processes, which partially act through mTOR pathway. At the same time, insulin removal and rapamycin could stimulate proteins synthesis that were related to cell death and growth phenotypes.

### Insulin/AKT-dependent translation inhibition is essential for cell survival

**Figure 2:**
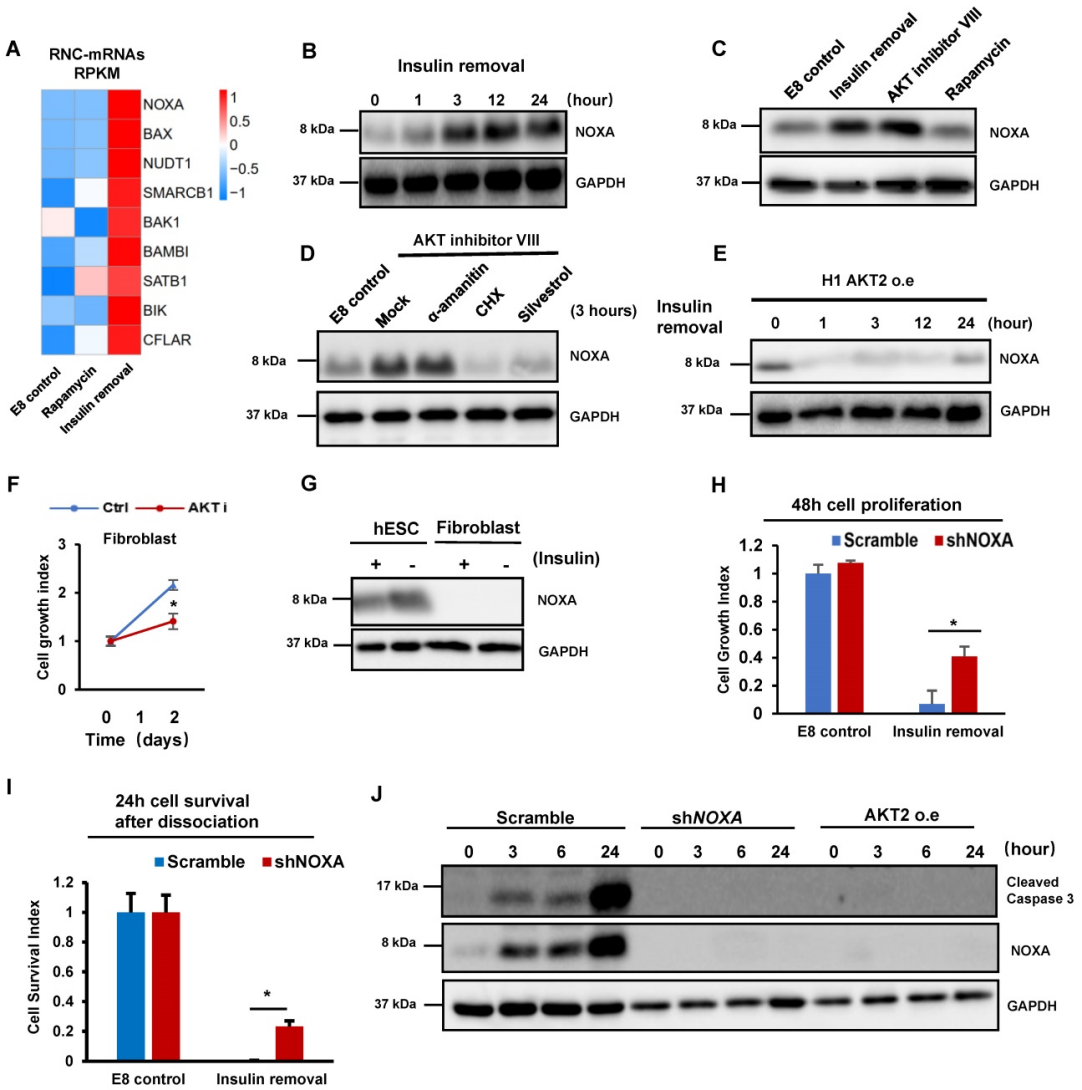
** Insulin/AKT-dependent translational inhibition of NOXA is essential for cell survival. (A)** Heatmap showing the levels of RNC-mRNAs of BH3-only members of the BCL-2 protein family in H1 cells under different treatments, including control (E8), insulin removal and rapamycin treatments. **(B)** Western blot showing protein levels of NOXA in H1 cells maintained in E8 without insulin for 0, 1, 3, 12 and 24 h. **(C)** Western blot showing protein levels of NOXA in H1 cells treated with indicated conditions for 1h. Insulin removal, E8 medium without insulin. AKT inhibitor VIII (10 μM). Rapamycin (100 nM). **(D)** H1 cells were cultured with indicated conditions for 3 h, western blot showing NOXA protein level. CHX, cycloheximide (100 μg/ml); eIF4A1 inhibitor silvestrol (25 μM); α-amatinin (10 μg/ml). **(E)** Western blot showing protein levels of NOXA in H1 cells with AKT overexpression maintained in E8 without insulin for 0, 1, 3, 12 and 24 h. **(F)** Growth curve of H1 cells and CCD-1139Sk fibroblasts maintained in E8 medium with or without AKT inhibitor VIII (10 μM) for 2 days. Cells were counted on day 0 and day 2 (n = 3 biological replicates). Cell counts were normalized to day 0 numbers. **(G)** Western blot showing the expression of NOXA in H1 cells and CCD-139Sk fibroblasts treated with or without insulin for 1 h in E8 medium. **(H)** 48-hour proliferation of H1 cells with NOXA knockdown (shNOXA) in E8 medium with or without insulin, compared to control (Scramble, shRNA with scrambled sequence). Cell counts are normalized to control cell count in E8 (n = 3, ∗, p < 0.05). **(I)** Survival numbers of H1 cells with NOXA knockdown (shNOXA) compared to control cells (Scramble) in E8 with or without insulin for 24 h. Cell counts were normalized to day 0 numbers (n = 3 biological replicates, ∗, p < 0.05). **(J)** Western blot showing levels of cleaved caspase 3 and NOXA in H1 cells with NOXA knockdown (shNOXA) cells compared to those in control cells (Scramble). Cells were maintained in E8 without insulin for 0, 3, 6, 24 hours.

Because cell death is induced only by insulin removal (Figure 1A), we hypothesized that insulin removal may upregulate ribosomal-associated mRNA to induce cell death through mRNA translation. Indeed, p53 pathway genes were increased in the ribosome-associated mRNAs after insulin removal according to the RNC-mRNA-Seq data (Figure S1C), including *BBC3, BAX, NOXA/PMAIP1, GADD45B* and *SESN3*. Interestingly, *BBC3, BAX* and* NOXA* all belong to the BH3-only subset of BCL-2 protein family. This family contains well-characterized apoptotic inducers, including *BAD, BIK/NBK, BIM/Bod, BMF, HRK/DP5, NOXA/PMAIP1* and *BBC3/PUMA.* Most of these genes showed enhanced ribosome-association only after insulin removal but not rapamycin treatment (Figure 2A and S2A). Among these genes, *NOXA* had the highest increase (more than four-fold) in ribosome-association within 2 hours upon insulin removal (Figure 2A). We examined whether ribosomal association level correlated with protein synthesis. Western blot showed that NOXA protein was indeed substantially increased after insulin removal (Figure 2B). Moreover, NOXA expression was suppressed by insulin in a dose-dependent manner (Figure S2B).

We then inspected which downstream pathways of insulin are responsible for NOXA regulation. We first demonstrated that NOXA translation was elevated by AKT Inhibition in one hour (Figure 2C). However, rapamycin treatment did not induce NOXA expression (Figure 2C), even after the treatment was extended to 24 hours (Figure S2C). AKT inhibition-induced NOXA synthesis was not inhibited by transcription inhibitor α-amanitin, but by translation inhibitor cycloheximide (CHX) and silvestrol (Figure 2D). Thus, translational regulation is a dominant factor for controlling NOXA gene expression in hPSCs. We further showed that the overexpression of constitutively active AKT2 blocked the increase in NOXA translation after insulin removal (Figure 2E). P53 is known to be the upstream of NOXA 26 , we checked the P53 protein level after insulin removal and AKT inhibition, while the result showed that the translation of P53 was not upregulated in the same way as NOXA (Figure S2D). Meanwhile, YBX1 has been reported as a negatively regulator of NOXA translation after AKT phosphorylation^27^, and western blot showed that insulin removal led to decreased YBX1 phosphorylation (Figure S2E). Polysome profiles showed that AKT inhibition increased the abundance of 80S monosomes and decreased polysomes in hESCs (Figure S2F), mimicking the effect of insulin removal (Figure 1D). These results support the notion that insulin regulate hPSC translation through AKT pathway. Although global translation was suppressed, insulin removal and AKT inhibition promoted specific mRNA translation involved in apoptosis.

Insulin is necessary for cell survival in hESCs, but not in fibroblasts 3, so we examined whether the cell type specific phenotype correlates with their sensitivity to AKT inhibition. We showed that AKT inhibition resulted in cell death in hESCs, however, it only led to growth arrest in fibroblasts without significant cell death (Figure 2F). We found that fibroblasts had significantly lower *NOXA* mRNA and protein level than hPSCs (Figure 2G and S2G). Therefore, we knocked down *NOXA* by shRNA in hPSCs (Figure S2H). Importantly, *NOXA* knockdown in hPSCs significantly improved cell survival in adherent cells in the absence of insulin (Figure 2H). *NOXA* knockdown even suppressed the cell death in individualized cells when insulin was not present after passaging (Figure 2I). Similarly, insulin also improved cell survival under AKT inhibitor treatment (Figure S2I). We then showed that *NOXA* knockdown suppressed the cleavage of caspase 3 after insulin removal (Figure 2J). These results indicated that insulin promotes cell survival by suppressing *NOXA* mRNA translation through AKT.

### Insulin removal and rapamycin induce eIF4E phosphorylation and growth arrest

**Figure 3:**
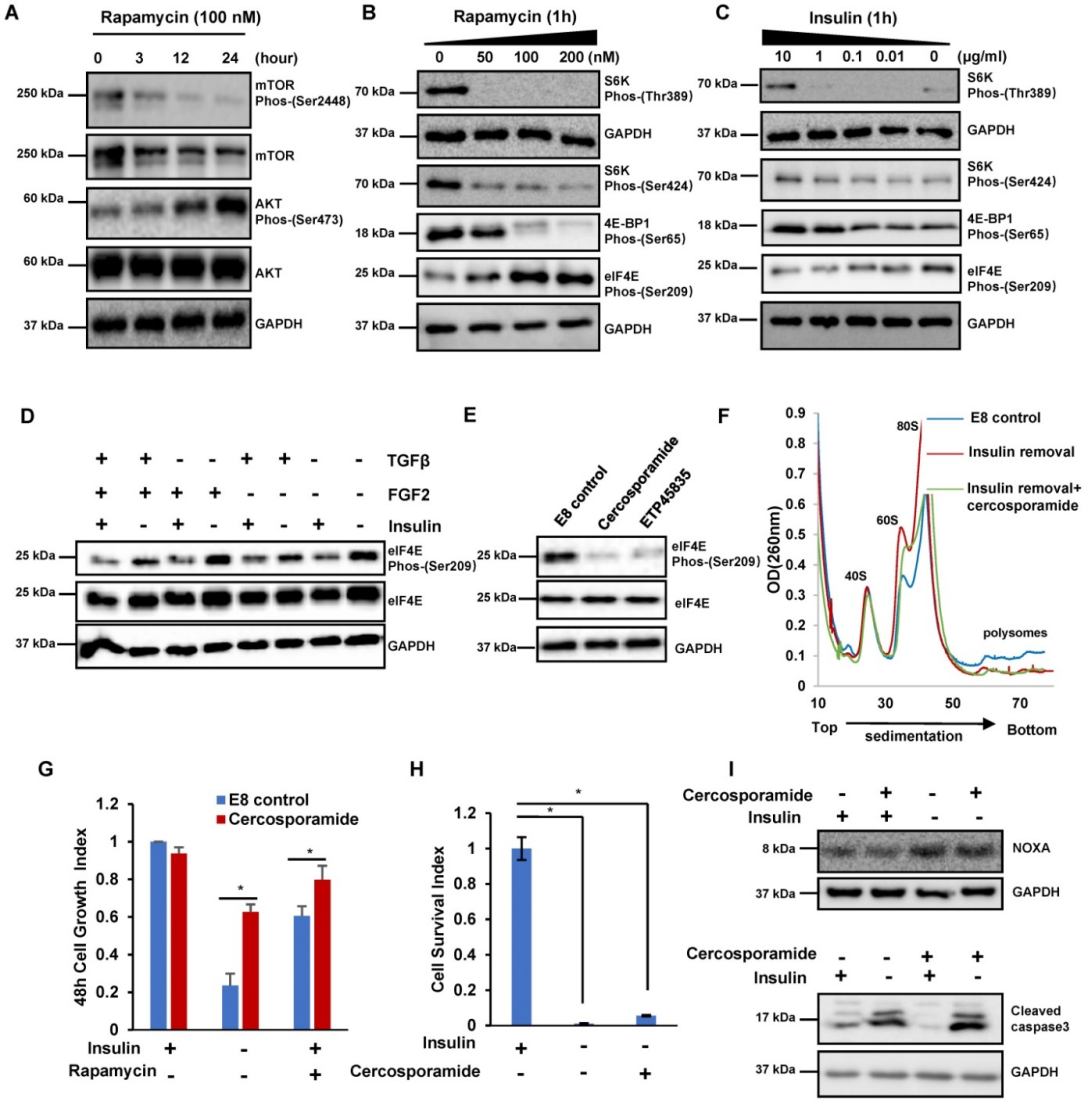
** The insulin/mTOR pathway inhibits eIF4E phosphorylation to control cell growth and translation. (A)** Western blot analysis of mTOR and AKT phosphorylation in H1 cells treated with rapamycin (100 nM) for 24 hours in E8. **(B-C)** Western blot analysis of eIF4E, S6K and 4E-BP1 phosphorylation levels in H1 cells treated with rapamycin or insulin at different concentrations for 1h. **(D)** Western blot showing protein levels of phospho-eIF4E and total eIF4E in H1 cells treated with or without specific mitogens commonly used for hESC maintenance. **(E)** Western blot analysis of eIF4E phosphorylation levels in H1 cells treated with indicated conditions. cercosporamide (1 μM); ETP45835 (10 μM). **(F)** Polysome profiles of H1 cells cultured with indicated conditions. (Data are representative of two independent experiments). **(G)** Effect of cercosporamide (1 μM) on H1 cell growth under insulin removal or rapamycin treatment. Cells were counted after 48 hours treatment. The cell growth index represents the cell number on day 2 divided by the cell number day 0 (n = 3 biological replicates, ∗, p < 0.05). **(H)** Effect of cercosporamide (1 μM) on H1 cell survival during passage without insulin. H1 cells were passaged into new plate with indicated conditions. The cell survival index represents the cell number at 24 hours divided by the cell number on day 0 (n = 3 biological replicates, ∗, p < 0.05). **(I)** Western blot showing protein levels of NOXA and cleaved caspase 3 treated under indicated conditions for 3 hours.

Similar to insulin removal, mTOR inhibitor rapamycin suppressed cell proliferation but it did not induce obvious cell death (Figure 1A). These data implicated that insulin may promote hESCs proliferation through mTOR-related mechanisms, and we further examined the potential downstream targets of insulin and mTOR. Rapamycin considerably reduced mTOR phosphorylation, but elevated AKT phosphorylation when the treatment was extended (Figure 3A). Within 1 hour, rapamycin decreased the phosphorylation of S6 and 4E-BP1 phosphorylation, however, it also significantly elevated eIF4E phosphorylation (Figure 3B). Similar to rapamycin, insulin removal also resulted in decreased S6K and 4E-BP1 phosphorylation but increased eIF4E phosphorylation (Figure 3C). The phosphorylation of S6K and 4E-BP1 is well known in mTOR-related translation regulation, however, the function of eIF4E phosphorylation has not been explored in hPSCs, so we focused on eIF4E phosphorylation here. Among all the mitogens essential for hESCs maintenance, insulin was the only factor whose removal led to eIF4E phosphorylation (Figure 3D). Together, these data strongly suggested that insulin acts through mTOR to suppress eIF4E phosphorylation in hPSCs.

Next, we further characterized the kinase cascades downstream of insulin. we found that both MNK inhibitor cercosporamide (Cerco) and ETP45835 (ETP) suppressed eIF4E phosphorylation in H1 cells (Figure 3E). This result is consistent with the previous report that MNK phosphorylates eIF4E 28. Therefore, we concluded that insulin likely suppresses eIF4E phosphorylation through insulin/mTOR/MNK pathway.

We further investigated the functional roles of eIF4E phosphorylation in hESCs. Inhibiting eIF4E phosphorylation by cercosporamide had negligible effect on polysome profile in E8 medium (Figure S3A). Cercosporamide did not significantly affect cell proliferation and pluripotency markers (Figure S3B and S3C). These data suggested that eIF4E phosphorylation level did not affect regular stem cell maintenance. We then examined how eIF4E phosphorylation affected hESCs during insulin removal and rapamycin treatment. Under insulin removal condition, cercosporamide decreased the amount of 60S and 80S monosomes but did not affect polysome abundance, implying that eIF4E phosphorylation had no effect on global translation (Figure 3F and S3D). We further showed that cercosporamide enhanced cell growth under insulin removal and rapamycin treatment (Figure 3G), however, cercosporamide could not sustain cell survival during passage (Figure 3H). Western blot showed that NOXA synthesis and Cleaved Caspase3 were not suppressed by cercosporamide after insulin removal, even though eIF4E phosphorylation was suppressed (Figure 3I). These results suggested that insulin modulates eIF4E phosphorylation to affect cell proliferation, but eIF4E phosphorylation is not involved in the translation of proapoptotic proteins, such as NOXA.

### hESC pluripotency is maintained under the mTOR inhibition by rapamycin

**Figure 4:**
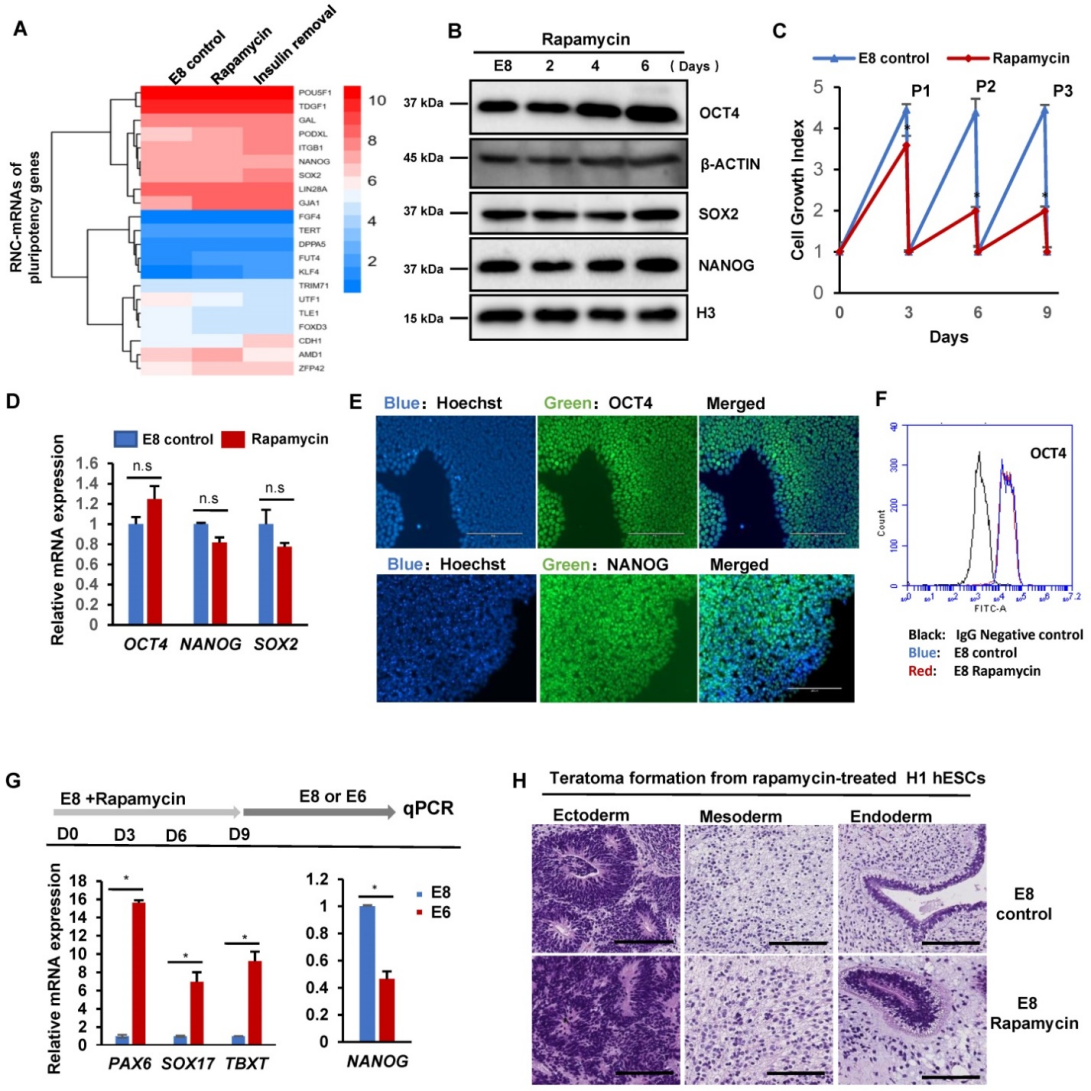
** mTOR-associated translation is not required for hESC pluripotency. (A)** Heatmap showing levels of RNC-mRNAs of pluripotency genes in H1 cells under specified treatments. **(B)** Western blot analysis of OCT4, SOX2 and NANOG in H1 cells cultured with or without rapamycin (100 nM) for 0, 2, 4 and 6 days. **(C)** Growth curve of H1 cells cultured with or without rapamycin (100 nM) in E8. Cells were passaged every 3 days and counted before and after passaging, and each time 5 x 10^4^ cells were seeded into one well of 12 well-plate. Numbers represent the total cell count in one well and are normalized to the day 0 numbers. (n = 3 biological replicates, ∗, p < 0.05). **(D)** Expression of pluripotency markers *OCT4, NANOG* and *SOX2* in H1 cells cultured with or without rapamycin (100 nM) for 3 passages, measured by qPCR. (n=3 biological repeats, n.s, non-significant). **(E)** Immunostaining of *OCT4* and *NANOG* in H1 cells cultured with or without rapamycin (100 nM) for 3 passages. Hoechst indicates nuclei. Scale bar, 200 μm. **(F)** FACS analysis of the OCT4+ population in H1 cells cultured in E8 medium with or without rapamycin (100 nM) for 3 passages. H1 cells incubated with second antibodies only was used as negative control. **(G)** Differentiation of rapamycin-treated hESCs. H1 cells cultured with or without rapamycin (100 nM) for 3 passages were switched to E6 medium (E8 without FGF2 and TGFβ) and allowed to spontaneously differentiation for 6 days before sample collection for qPCR analysis. (n = 3 biological repeats and are representative of 3 independent experiments, ∗, p < 0.05).Pluripotency marker: *NANOG*. Ectoderm marker: *PAX6*. Mesoderm marker: *TBXT*. Endoderm marker: *SOX17*. **(H)** Histological examinations of teratomas formed by hESCs pretreated with rapamycin (100 nM) for 3 passages, showing the presence of tissues from all three germ layers. Scale bar, 200 μm.

Rapamycin leads to paused pluripotency in mouse 18, but it induces hPSC differentiation in KOSR-containing maintenance medium 20. We investigated how translation inhibition influences pluripotency in hPSCs. Based on our RNC-seq data, we found that there was little change of ribosome-associated mRNAs in the pluripotency marker genes after 2-hour rapamycin treatment in chemically defined E8 medium (Figure 4A). It suggests that rapamycin-induced translational inhibition does not lead to immediate translation response to exit from pluripotency. When rapamycin was applied to hPSCs for 6 days, no significant changes were observed in key pluripotency proteins (Figure 4B). This data suggested that mTOR inhibition by rapamycin probably do not have major influence on the translation of key pluripotency proteins.

We further showed that hPSCs could be maintained in E8 with rapamycin for multiple passages with reduced proliferation rate (Figure 4C), but there were no significant morphology changes (Figure S4A). Meanwhile, the mRNA levels of pluripotency genes, including *OCT4*, *NANOG* and *SOX2*, were well maintained (Figure 4D). The expression of pluripotency markers was confirmed with immunostaining and flow cytometry analysis (Figure 4E-F). We then demonstrated that pluripotency genes expressed in H9 hESC and NL-4 iPSC lines under rapamycin treatment in E8 medium (Figure S4B). Functionally, hPSCs cultured with rapamycin for 3 passages were fully capable to spontaneously differentiated into endoderm, mesoderm and ectoderm lineages *in vitro* in the E6 medium (E8 medium without FGF2 and TGFβ) (Figure 4G). They also formed teratomas in immunodeficient mice and formed all three germ layers (Figure 4H and S4C). These data indicated that mTOR inhibition by rapamycin suppresses hPSC proliferation without affecting pluripotency in chemically defined medium.

We then investigated why rapamycin was observed to induce differentiation in KOSR-containing culture 20, but not in E8 medium. We showed that rapamycin did not significantly induce differentiation in the E8 medium, but elevated levels of ectoderm (*PAX6*) and mesoendoderm (*SOX17*) markers were observed after 6-day treatment in E8 supplemented with KOSR (Figure S4D). KOSR contains differentiation-inducing contaminants that could activate BMP pathway 29, 30. When BMP4 was present, rapamycin potentiated mesoendoderm differentiation (Figure S4E). Under ectoderm differentiation condition (SB431542), rapamycin also enhanced ectoderm induction (Figure S4E). These data suggested that rapamycin could potentiate differentiation under contaminated inducers in KOSR. Therefore, these results supported our model that mTOR inhibition by rapamycin does not cause the loss of pluripotency but enhances hPSC sensitivity to differentiation stimuli.

### mTOR inhibition induces unique gene expression in dormant pluripotency

**Figure 5:**
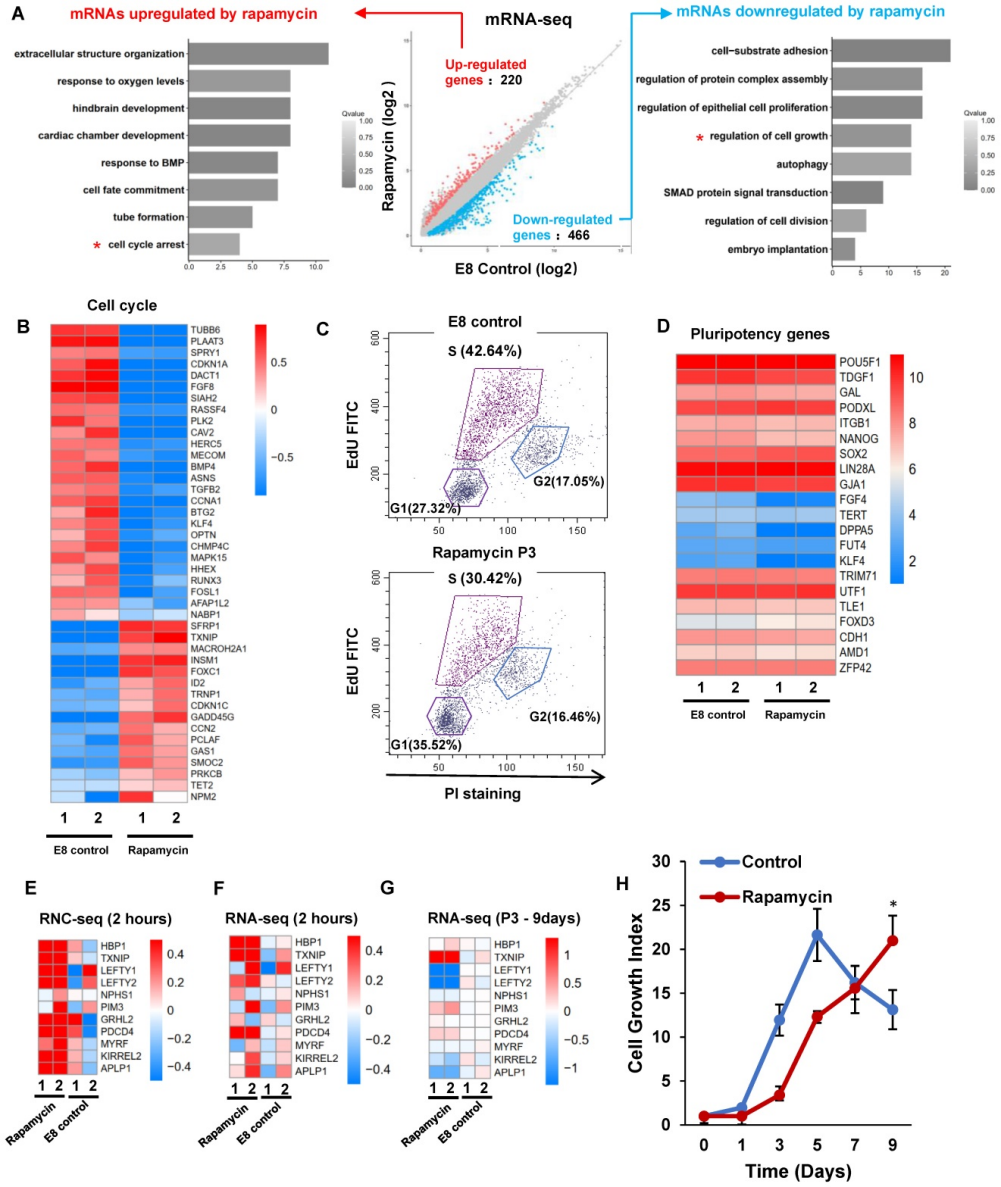
** mTOR inhibition induces dormancy gene signature in hESC. (A)** H1 cells were cultured in E8 control or E8 with rapamycin (100 nM) for 3 passages. Scatter plot and GO terms showing differentially expressed genes. **(B)** Heatmap showing the rapamycin regulated differentially expressed genes associated with the cell cycle (GO:0007049). **(C)** Cell-cycle profiles of hESCs maintained in E8 or E8 with rapamycin (100 nM) for 3 passages. EdU (5-ethynyl-2′-deoxyuridine) and Propidium Iodide (PI) were used to do cell cycle analysis. **(D)** Heatmap of the pluripotency marker genes in hESCs maintained in E8 or E8 with rapamycin (100 nM) for 3 passages. **(E-G)** Heatmap showing levels of indicated genes sets under E8 control, rapamycin(2h) or rapamycin (3 passages) treatments. (E) and (G) are RNA seq data. (F) is RNC-RNA seq data. **(H)** Growth curve of hESCs with or without rapamycin (100 nM). E8 medium was changed every day. Cells were counted every 2 days. (n = 3 biological replicates, ∗, p < 0.05).

Under rapamycin treatment, hPSCs maintain pluripotency with slower proliferation rate. We analyzed global gene expression with RNA-seq on hPSCs treated with rapamycin for three passages (Figure 5A). Rapamycin upregulated 220 genes, and downregulated 466 genes. The upregulated genes were enriched in cell cycle arrest, responses to oxygen level and BMP; while the downregulated genes were enriched in cell growth, proliferation, and cell adhesion. Of the rapamycin-regulated genes, 42 were involved in cell cycle regulation (GO:0007049) (Figure 5B). Noticeably, CDKN1C and GADD45G were elevated by rapamycin, which are known in cell cycle arrest 31, 32. Meanwhile, 59 proliferation-associated genes (GO:0008283) and 18 growth related genes (GO:0016049) were differentially regulated by rapamycin (Figure S5A and S5B). We then showed that rapamycin decreased S phase cell population while increased G1 phase cells (Figure 5C). These data suggested that rapamycin altered cell cycle profile at least partially through transcriptional regulation.

We then showed that the pluripotency genes expression was not significantly decreased by rapamycin (Figure 5D), and lineage markers were not elevated (Figure S5C). Meanwhile, rapamycin modulated the expression of primed stage marker genes (Figure S5D), but it had no significant influence to induce naïve stage markers (Figure S5E). We then compared short-term and long-term transcriptome responses to rapamycin. 2-hour rapamycin treatment elevated the gene expression associated with diapause state that was reported in mESCs 18 (Figure 5E-F). However, most of those changes were not obvious after rapamycin were applied for 3 passages (Figure 5G). These results suggested that extended rapamycin treatment induced dormant hPSCs at the primed state, which are different from dormant pluripotent cells of naïve mESCs.

Finally, we explored how rapamycin-induced dormant state could be used in stem cell applications. The rapid proliferation of hPSCs demands daily medium change and frequent expansion that require a lot of resources, otherwise cells would be damaged by lactic acidosis 33. Severe cell death was observed in cells without passage even medium was replenished daily, however, rapamycin helped maintain a slower but steady growth without massive death (Figure 5H and S5F). We also showed that rapamycin allowed continuous proliferation without medium change in comparison with regular E8 (Figure S5G). These data indicated that mTOR inhibition could help hPSC maintenance at suboptimal conditions, which would be useful in future stem cell applications such as cell expansion and transportation.

## Discussion

In hPSCs, thousands of cellular processes are simultaneously carried out to sustain self-renewal and pluripotency. A handful of mitogens are sufficient to achieve a signaling balance necessary for regular maintenance, so each mitogen needs to direct multiple processes in concert. As the final stage of central dogma, translational regulation provides an ideal platform for mitogens to influence various cellular functions by tuning distinctive protein production. Insulin exemplifies the importance of multiplex translational regulation in hPSC maintenance. Insulin is required for not only the activation of substrate-specific translation but also the translational inhibition of proteins deleterious to cells. Such dichotomous regulation allows insulin to promote cell survival and proliferation concurrently. The understanding of these fundamental mechanism would greatly enhance our ability to develop new stem cell applications through mitogenic pathways.

Similar to somatic cells, insulin activates PI3K/AKT/mTOR axis to promote mRNA translation in hPSCs. Insulin removal or mTOR inhibition leads to substantial increase of free ribosome and monosomes, implying the suppression of global mRNA translation. Interestingly, both insulin removal and rapamycin increase eIF4E phosphorylation that contributes to lower global mRNA translation. The inhibition of eIF4E phosphorylation partially rescues hESC growth under insulin removal and rapamycin. eIF4E phosphorylation compromises the stability of interaction between the eIF4F complex and mRNA thus leading to decreased protein synthesis 34. Rapamycin-induced eIF4E phosphorylation was also found to regulate translation in cancer cells 35-38. It would be interesting to explore whether a common mechanism is utilized by both cancer cells and stem cells. Meanwhile, it is well known that mTORC1 and mTORC2 are involved in mTOR pathway regulation. It is important to understand how these two distinct complexes regulate eIF4E phosphorylation and mRNA translation in future studies.

Besides promoting mRNA translation, insulin and mTOR also inhibits translation that are deeply involved in hPSC maintenance. While insulin removal and rapamycin decrease hundreds of ribosome-associated mRNAs, they simultaneously enhance another set of ribosome-associated mRNAs with similar magnitude. Insulin-dependent inhibition is particularly important for hPSC survival. Insulin activates AKT to suppress the production of pro-apoptotic proteins such as NOXA. NOXA suppression rescues hPSC survival in the absence of insulin. The insulin/AKT associated NOXA inhibition is the opposite from the observation in somatic cells. In somatic cells and cancer, *NOXA* transcription and later protein synthesis are dependent on PI3K/AKT, which implying that AKT pathway somewhat contributes to NOXA-related cell death in somatic cells 39, 40, 41. The drastic cell type specificity of NOXA synthesis and cell death suggest that the AKT dependence diminishes after differentiation. It is still to be explored how translation regulation transition take place during the exit from pluripotency.

Although both AKT and mTOR are insulin's downstream effectors in translation regulation, their modulations lead to distinctive RNC-mRNA profiles. Similar to AKT, differential translation regulation could exist to other signal transducers either upstream or downstream mTOR. Such regulatory ladder greatly expands a signaling pathway's potential to multitask in many biological processes. If transcriptome impact is also considered, a single mitogen's regulation could be expanded even more. It is intriguing to examine the coordination of transcription and translation in stem cell maintenance and differentiation.

When hPSC translation is inhibited by rapamycin in chemically defined E8 medium, the primed pluripotency is maintained at a dormant state. Cells at dormant state do not need frequent medium changes and passaging, and beneficial for cell maintenance at suboptimal condition. Cells can regain proliferative ability when rapamycin is removed. Although dormant state is achievable in both naïve and primed states, transcriptome changes of dormant hPSCs are significantly different from dormant mESCs at naïve state. The molecular mechanism of cell type specific dormant pluripotency is still to be explored.

In summary, insulin directs dichotomous regulation on hESC translation to promote cell survival and proliferation. Such translation regulation could be a common format for a single mitogen to coordinate various biological processes at the same time. When multiple mitogens are involved, they are able to greatly enhance the regulatory complexity to either maintain pluripotency or induce differentiation.

## Supplementary Material

Supplementary figures.

Supplementary table 1.

Supplementary table 2.

Supplementary table 3.

## Figures and Tables

**Table 1:** Primers used for real-time PCR

Gene	Primer	Sequence
*OCT4*	OCT4-F	AGCGAACCAGTATCGAGAACC
	OCT4-R	CTGATCTGCTGCAGTGTGGGT
*NANOG*	NANOG-F	TTTGTGGGCCTGAAGAAAACT
	NANOG-R	AGGGCTGTCCTGAATAAGCAG
*SOX2*	SOX2-F	AGTGTTTGCAAAAGGGGGAAAGTAG
	SOX2-R	CCGCCGCCGATGATTGTTATTATT
*PAX6*	PAX6-F	CACCTACAGCGCTCTGCCGC
	PAX6-R	CCCGAGGTGCCCATTGGCTG
*SOX17*	SOX17-F	CGCACGGAATTTGAACAGTA
	SOX17-R	GGATCAGGGACCTGTCACAC
*TBXT*	TBXT-F	CCCTATGCTCATCGGAACAA
	TBXT-R	CAATTGTCATGGGATTGCAG
*NOXA*	NOXA-F	ACCAAGCCGGATTTGCGATT
	NOXA-R	ACTTGCACTTGTTCCTCGTGG

## Abbreviations

hPSCs: human pluripotent stem cells
hESC: human embryonic stem cell
iPSC: induced pluripotent stem cell
eIF4F: eukaryotic initiation factor 4F complex
mTOR: mammalian target of rapamycin
MAPK: mitogen-activated protein kinase
S6K: ribosomal protein S6 kinase
MNKs: MAPK interacting protein kinases
KOSR: Knock-Out Serum Replacement
RNC: ribosome-nascent chain complex
GO: Gene Ontology
SRA: Sequence Read Archive
Cerco: cercosporamide
ETP: ETP45835
CHX: cycloheximide
