# Supplementary material for: Insulin Directs Dichotomous Translational Regulation to Control Human Pluripotent Stem Cell Survival, Proliferation and Pluripotency
Source: Int J Biol Sci. 2022 May 16;18(9):3562–75. doi: 10.7150/ijbs.71199 (PMC9254461; doi:10.7150/ijbs.71199)
Supplement: Supplementary file 1 — Supplementary figures. [file ijbsv18p3562s1.pdf]

Figure S1

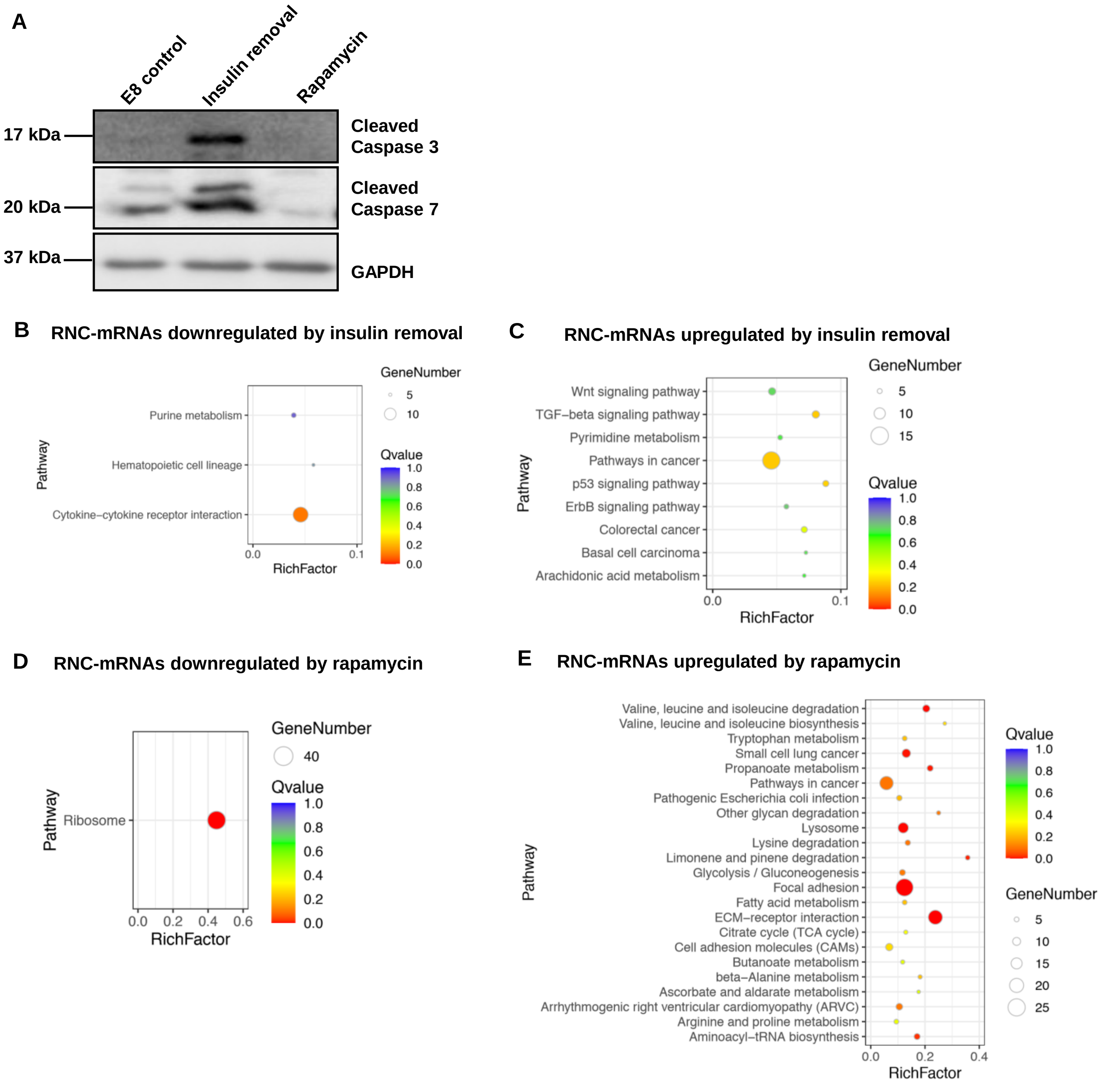

Figure S1. **Related to figure 1.**

Divergent translational regulation by insulin through AKT and mTOR.

(A) Western blot showing protein levels of cleaved caspase3/7 in H1 cells treated with indicated conditions for 24 hours. GAPDH is used as loading control.

(B-E) KEGG pathway analysis of genes modulated by insulin removal or rapamycin treatment.

Figure S2

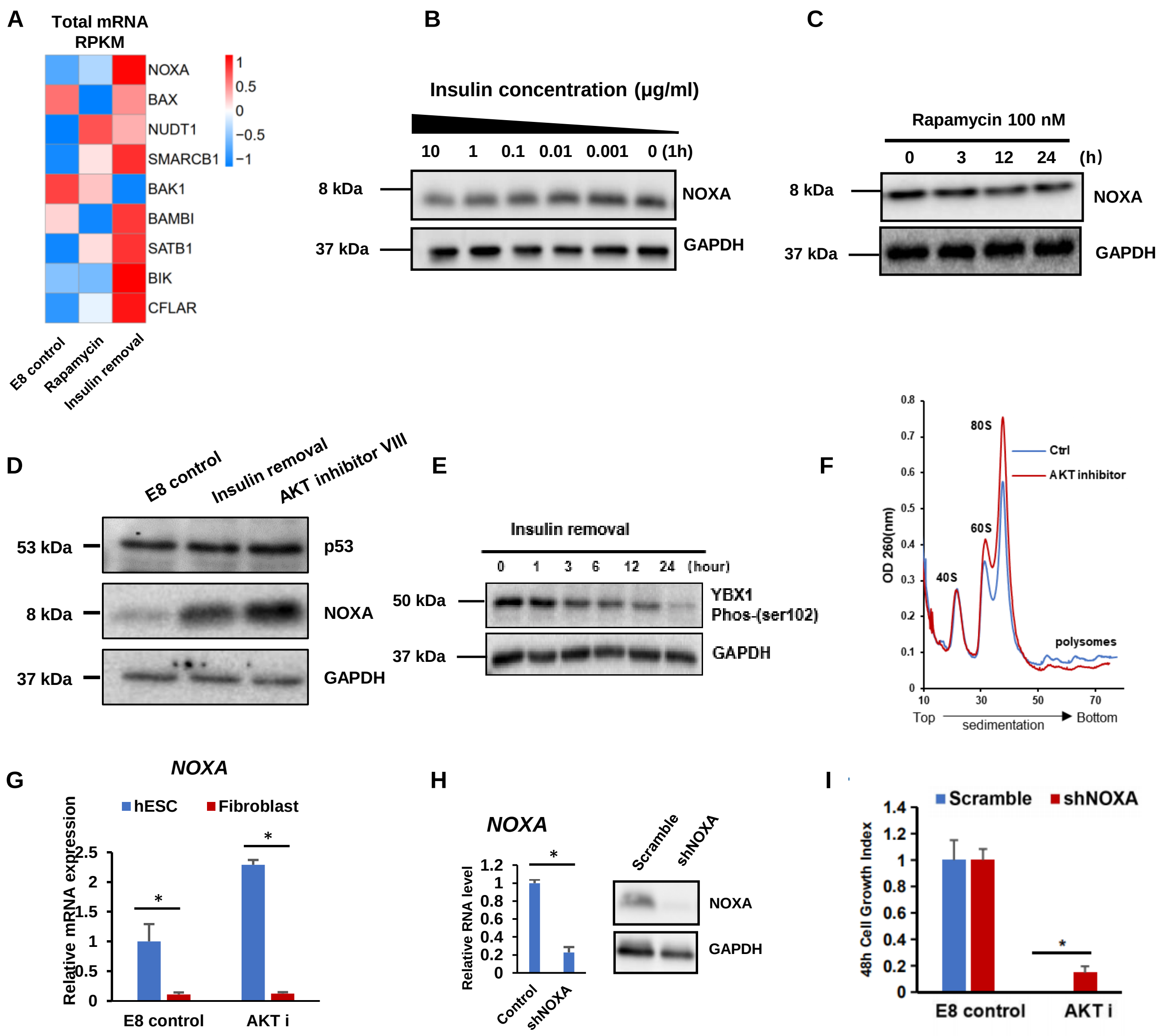
A
Total mRNA RPKM
NOXA
BAX
NUDT1
SMARCB1
BAK1
BAMBI
SATB1
BIK
CFLAR
1
0.5
0
−0.5
−1
E8 control
Rapamycin
Insulin removal
B
Insulin concentration (μg/ml)
10 1 0.1 0.01 0.001 0 (1h)
8 kDa
NOXA
37 kDa
GAPDH
C
Rapamycin 100 nM
0 3 12 24 (h)
8 kDa
NOXA
37 kDa
GAPDH
D
E8 control
Insulin removal
AKT inhibitor VIII
53 kDa
p53
8 kDa
NOXA
37 kDa
GAPDH
E
Insulin removal
0 1 3 6 12 24 (hour)
50 kDa
YBX1 Phos-(ser102)
37 kDa
GAPDH
F
OD 260(nm)
80S
60S
40S
Ctrl
AKT inhibitor
polysomes
Top
sedimentation
Bottom
G
NOXA
hESC
Fibroblast
Relative mRNA expression
E8 control
AKT i
H
NOXA
Relative RNA level
Control
shNOXA
Scramble
shNOXA
NOXA
GAPDH
I
Scramble
shNOXA
48h Cell Growth Index
E8 control
AKT i

Figure S2. Related to figure 2.

Insulin/AKT pathway inhibits NOXA translation.

(A) Heatmap showing the total RNA levels of BH3-only member of the Bcl-2 family proteins in H1 cells under indicated treatment.

(B) Western blot showing protein levels of NOXA in H1 cells treated with different concentration of insulin for 1h.

(C) Western blot showing protein levels of NOXA in H1 cells treated with rapamycin for 0, 3, 12 and 24 h.

(D) Western blot showing protein level of P53 and NOXA in H1 cells following insulin removal and AKT inhibitor treatment for 3h.

(E) Western blot showing YBX1 phosphorylation in H1 cells at different time points following insulin removal.

(F) Polysome profiles of H1 cells treated with or without AKT inhibitor VIII (10 μM) for 2h.

(G) qPCR showing NOXA mRNA expression in H1 cells and CCD-1139Sk fibroblasts treated with or without AKT inhibitor VIII for 2h.

(H) Real time PCR (left) and western blot (right) analyses showing knockdown of NOXA in H1 cells with shRNA. H1 cells were transfected with shRNA-NOXA or shRNA-scramble as control(n = 3 independent experiments, $*$, $p < 0.05$).

(I) 48-hour survival of H1 cells with NOXA knockdown (shNOXA) treated with or without AKT inhibitor VIII in E8 medium, compared to control (Scramble, shRNA with scrambled sequence). Cell counts are normalized to control cell count in E8 without AKT inhibitor (n = 3 biological experiments, $*$, $p < 0.05$).

Figure S3

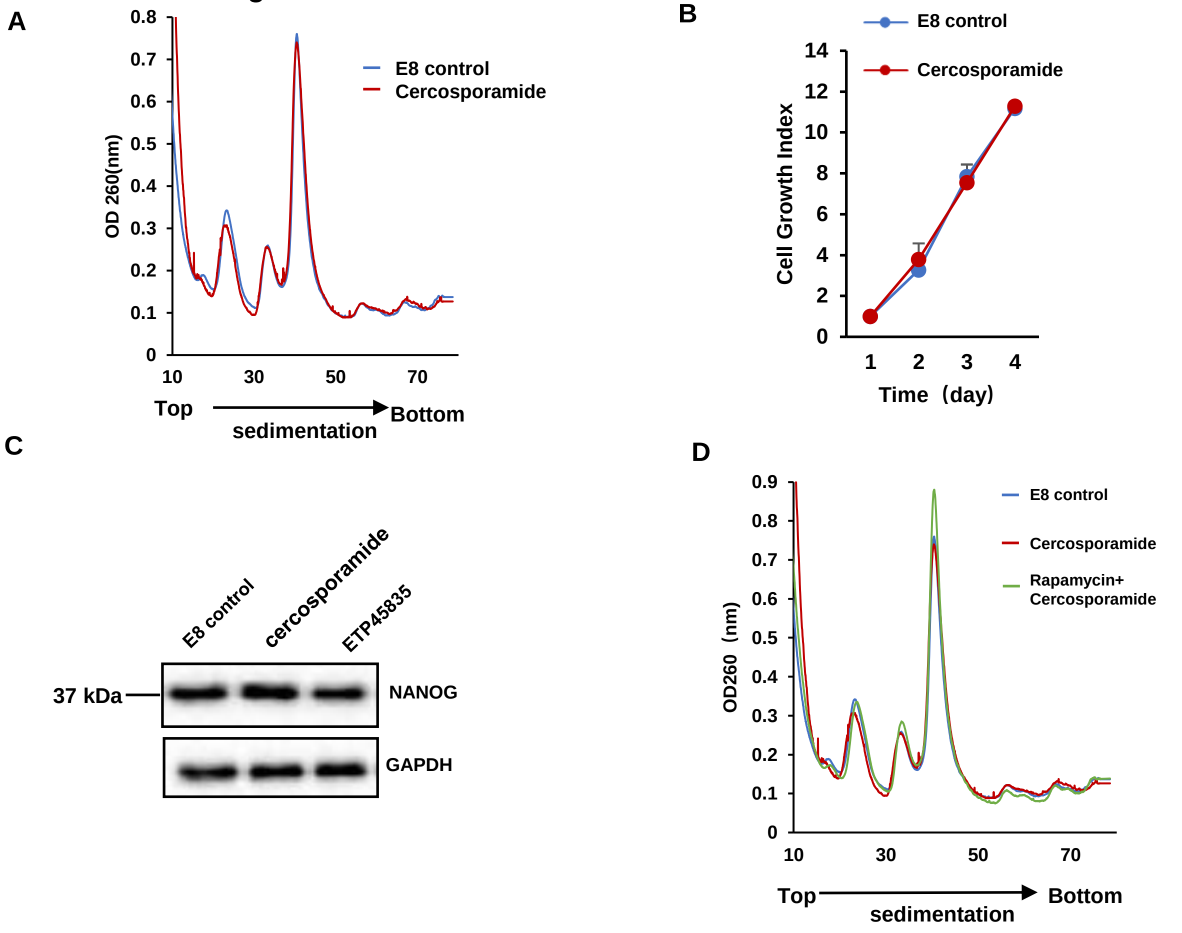

Figure S3. Related to figure 3.

The insulin/mTOR pathway inhibits eIF4E phosphorylation to control cell growth and translation.

(A) Effect of 2 hours cercosporamide (1 μM) treatment on polysome profiles of H1 cells.

(B) Cell number were counted every day during hESCs were cultured in E8 or cercosporamide (1 μM).

(C) Western blot showing NANOG expression of hESCs which were treated with indicated conditions for 24h.

(D) Polysome profiles of H1 cells treated with indicated conditions for 2 hours.

Figure S4

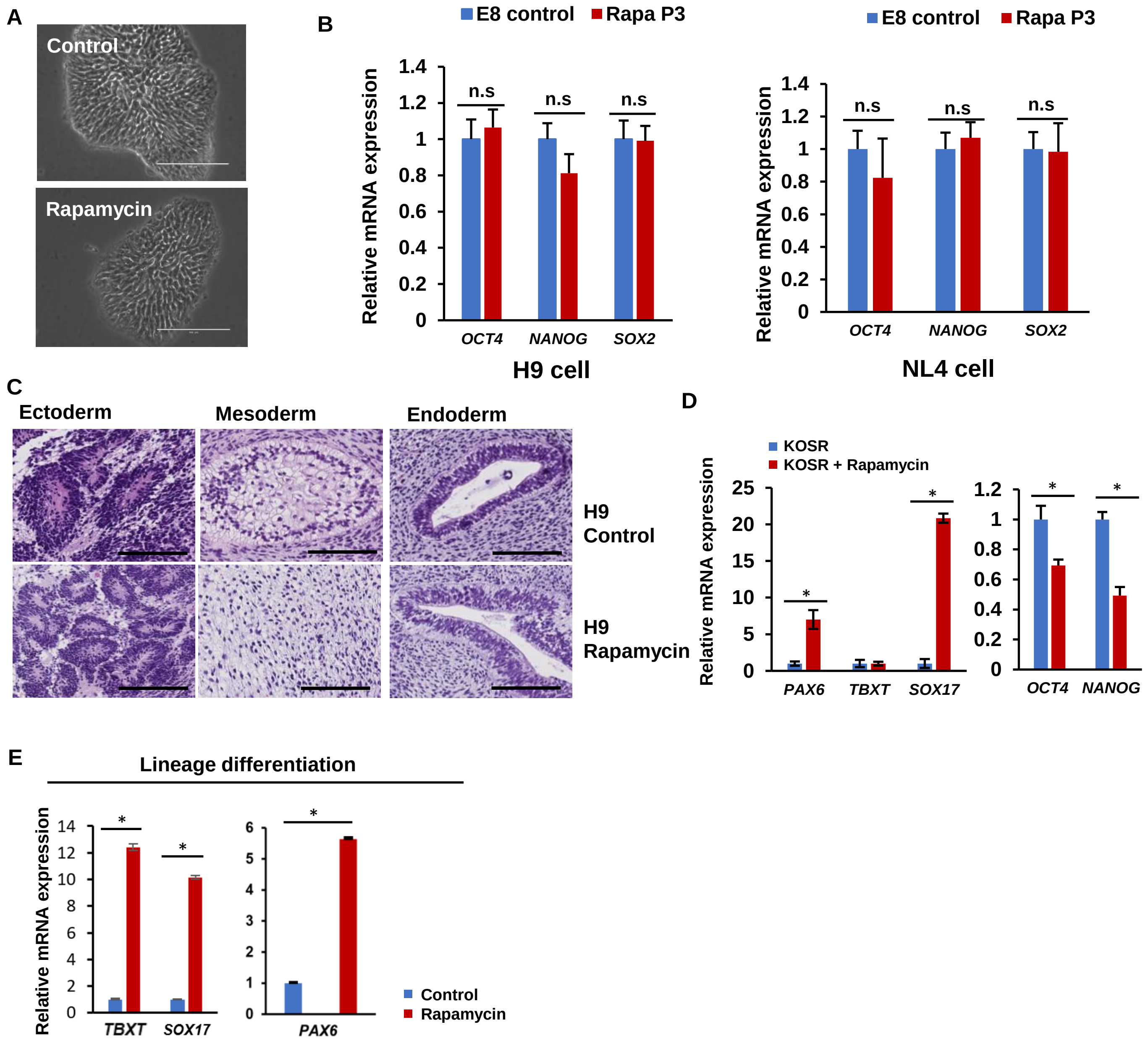

Figure S4. Related to figure 4.

mTOR-associated translation is not required for hESC pluripotency.

(A) Phase contrast image of H1 cells cultured with or without rapamycin (100 nM) for 3 passages. Scale bar, 100 µm.

(B) qPCR analysis of pluripotency markers in H9 and NL4 cells cultured with or without rapamycin (100 nM) for 3 passages. (n = 3 biological repeats . n.s: non-significant).

(C) H9 were cultured in E8 or rapamycin (100 nM) for 3 passages. cells were injected into Nude mice. H&E stained images of paraffin embedded teratomas show the presence of tissues from all three germ layers.

(D) qPCR analysis of pluripotency (*OCT4* and *NANOG*),mesoderm (*TBXT*), endoderm (*SOX17*) and ectoderm (*PAX6*) marker genes in H1 cells cultured in KOSR medium (DMEM/F12 with 20% KOSR) with the presence or absence of rapamycin (100nM). (n = 3 independent experiments, ∗, $p < 0.05$).

(E) qPCR analysis of mesoderm (*TBXT*), endoderm (*SOX17*) and ectoderm (*PAX6*) marker genes in H1 cells subjected to lineage-specific differentiation with the presence or absence of rapamycin (100nM) under BMP4 or SB431542 culture condition. (n = 3 biological repeats. ∗, $p < 0.05$).

Figure S5

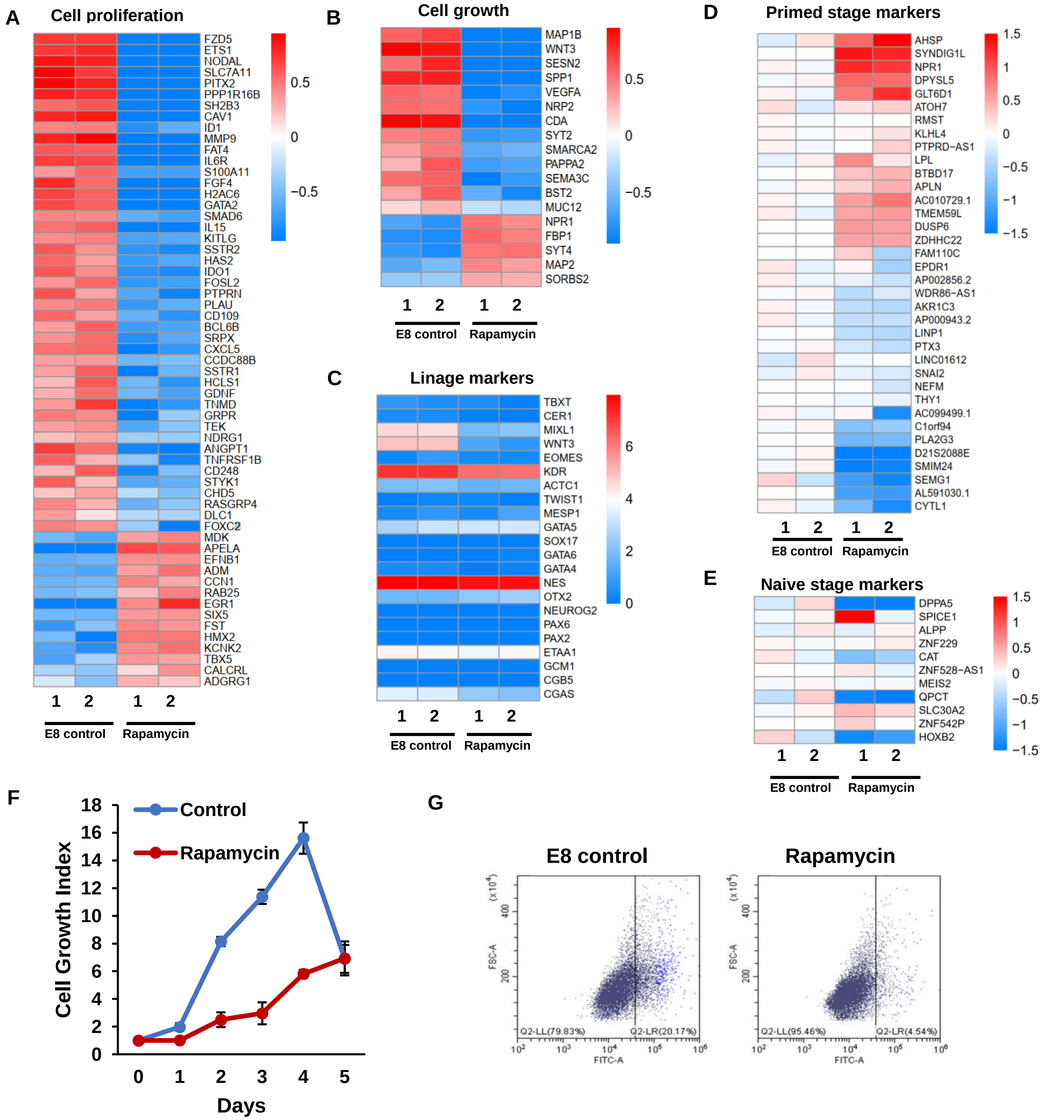

Figure S5. Related to figure 5.

Rapamycin long term treatment induces dormant hESC.

(A-B) Heatmap showing the rapamycin (100 nM) regulated differentially expressed genes associated to the cell proliferation (GO:0008283) and cell growth (GO:0016049).

(C) Heatmap representation of the commitment genes in hESC treated with or without rapamycin (100 nM).

(D-E) Heatmap representation of the primed and naive stage markers as defined by Tobias Messmer et al.(2019).

(F) Five-day culture of hESCs with or without rapamycin (100 nM). No medium change. Cells were counted every day. (n = 3 biological replicates).

(G) H1 Cells were cultured in E8 medium with or without rapamycin (100 nM) till cell density was 90%. CellEvent Caspase-3/7 Green detection reagent (C10423,Thermo) was added into medium for 30 mins before cells collection.
